# Prospective Monitoring of Newly Marketed Drugs in Frail Older Adults Using Real-World Databases

**DOI:** 10.1093/geroni/igab046.801

**Published:** 2021-12-17

**Authors:** Dae Kim, Elisabetta Patorno

## Abstract

In recent years several new drugs have been approved for treatment of heart failure and type 2 diabetes. Despite their life-prolonging benefits, uptake of new drugs is often slow among older patients with frailty due to under-representation of frail older adults in pivotal clinical trials and concerns for adverse events. To optimize pharmacotherapy, timely evaluation of the drug benefits and risks is urgently needed. We propose a novel drug monitoring framework that prospectively evaluates the effectiveness and safety of newly marketed drugs for frail and non-frail patients in real-world databases. This framework utilizes a validated claims-based frailty index (CFI) (range: 0-1; frail if ≥0.20) to find early signals for effectiveness and safety of new drugs by updating the analysis at regular intervals as new data become available. In this symposium, we present early results of this prospective monitoring framework for 2 new drug classes using Medicare claims data from the approval date until the end of 2017: 1) angiotensin receptor-neprilysin inhibitor (ARNI) (approved in July 2015) for heart failure with reduced ejection fraction (HFrEF) and 2) sodium-glucose cotransporter-2 inhibitors (SGLT2i) (approved in March 2013) for type 2 diabetes. We first show the uptake of ARNI and SGLT2i over time among the eligible Medicare beneficiaries by clinical characteristics, including frailty. Subsequently we present the results of sequential cohort analysis for the effectiveness and safety results of ARNI and SGLT2i. After these presentations, the panel will discuss the strengths, limitations, and challenges of implementing our monitoring framework in real-world databases.

